# Comparing Tactical Behaviour of Soccer Players in 3 vs. 3 and 6 vs. 6 Small-Sided Games

**DOI:** 10.2478/hukin-2014-0047

**Published:** 2014-07-08

**Authors:** Bernardo Silva, Júlio Garganta, Rodrigo Santos, Israel Teoldo

**Affiliations:** 1 Faculty of Sport, University of Porto, Porto, PORTUGAL.; 2 Centre of Research and Studies in Soccer - Universidade Federal de Viçosa, Viçosa, MG - BRAZIL.

**Keywords:** Soccer, tactical assessment, tactical behaviour, small-sided games

## Abstract

The present study aimed to compare players’ tactical behaviour in 3 vs. 3 and 6 vs. 6 soccer small-sided games (SSGs). The sample comprised 3,482 tactical actions performed by 18 U-11 youth soccer players from a Portuguese club, in 3 vs. 3 and 6 vs. 6 SSGs. All participants played eight minutes in both situations and field size was adapted according to the number of players involved (30 m × 19.5 m for 3 vs. 3 and 60 m × 39 m for 6 vs. 6). The System of Tactical Assessment in Soccer (FUT-SAT) was used for data collection and analyses. Descriptive analysis was conducted to verify frequencies and percentages of the variables assessed. The chi-squared (χ^2^) test was performed to compare the frequencies of the variables between 3 vs. 3 and 6 vs. 6 SSGs and Standardized Residuals (e) were used to examine the influence of the frequency of one or more variables within 3 vs. 3 and 6 vs. 6 SSGs. Data treatment was performed through SPSS for Windows®, version 18.0. Results indicated that players displayed safer behaviours in 6 vs. 6 SSG and more aggressive behaviours in 3 vs. 3 SSG. Findings can aid coaches and teachers to develop different players’ tactical skills according to the chosen SSG (3 vs. 3 or 6 vs. 6) form.

## Introduction

The study of team sports through the observation of behaviour patterns of players and teams is not recent, having occurred alongside with the constraints of expertise (Garganta, 2001). In soccer, as well as in other team sports, tactical behaviour can be defined as the sequences of actions performed by players aiming to deal, by the most appropriate means, with match situations, considering the constraints of time, space and task (Boulogne, 1972). Accordingly, the analysis of tactical behaviour should not be solely based on a particular action performed in isolation, but rather on general tactical patterns, which comprise all the typical characteristics of such isolated actions performed by all players within a team (Mahlo, 1969).

In this respect, tactical behaviour analyses in soccer have been conducted in recent years with the purpose of verifying to what extent this variable could be affected by other elements (Sampaio and Maçãs, 2012). Some researchers aimed to examine the association between tactical behaviour and contextual variables (i.e. match location, positional demands, match status and substitutions) or psychological features (i.e. motivation), and verified that players’ behaviour is likely to be influenced by these constraints to some point (Lago-Peñas, 2009; Myers, 2012; Shafizadeh and Gray, 2011; Taylor et al., 2004; Taylor et al., 2008). Other authors addressed the subject from the perspective of the impact that relative age effect (RAE) and changes in playing area might exert, with the role of RAE proving to be rather ineffective, while the increase or decrease of field size demonstrated that tactical behaviour patterns might undergo modifications according to spatial constraints (Dellal et al., 2011a; Teoldo et al., 2010b). While all the above-mentioned studies have been conducted in order to enhance general knowledge of tactical behaviour, there seems to be a lack of further research in this topic from the perspective of small-sided games (SSGs) and the variations in their structure, specially those regarding the number of players (Aguiar et al., 2012).

Youth soccer players have the need to foster numerous motor abilities as well as technical and tactical skills in order to attain higher levels of performance. Such development is dependent on exercise intensity and also on activities that enable players to communicate with each other and experience appropriate time in contact with the ball (Reilly, 2005; Silva et al., 2011). In order to attain all these goals, coaches rely on the use of small-sided games (SSGs) within the training process, since these structures seem to involve the necessary constraints to provide players with sufficient stimuli for the improvement of their performances (Almeida et al., 2013; Casamichana et al., 2012). Apparently, only a limited number of research has focused on the effects of modifications in SSGs over players’ tactical behaviour and more investigation appears to be necessary to enhance the current knowledge over this subject (Teoldo et al., 2010a; Teoldo et al., 2010b).

Therefore, the aim of this study was to compare players’ tactical behaviour in 3 vs. 3 and 6 vs. 6 soccer small-sided games (SSGs)..

## Material and Methods

### Sample and Participants

The sample comprised 3,482 tactical actions performed by 18 U-11 youth soccer players from a Portuguese club, in 3 vs. 3 and 6 vs. 6 small-sided games (SSGs). Players performed 1,787 actions in the 3 vs. 3 situation and 1,695 in 6 vs. 6. The actions in which players performed throw-ins, free kicks, corner-kicks, as well as those, in which they did not perform any tactical actions, were not considered for assessment.

The club signed a Statement of Authorization, allowing researchers to test the players of the corresponding academy level as well as to utilize its facilities for the conduction of the tests. Parents or guardians signed a written informed consent form, authorizing players to take part in the research.

This research had the approval of the Ethics Committee from the University of Porto, Portugal (CEFADE 15/2013) and meets the standards of the Declaration of Helsinki for research with human beings (1996).

### Procedures

Data were collected with the permission of club’s representatives. Players were informed about the objectives of the research and also about the purposes of the tests they were about to perform. Players did not attend training sessions on test days to avoid physical and cognitive strain, which could affect their performance during the tests. All participants played during eight minutes in both situations (3 vs. 3 and 6 vs. 6). Playing area was adapted according to the number of players involved, and in the 3 vs. 3 field size was 30 m long and 19.5 m wide, while in the 6 vs. 6 it was 60 m long and 39 m wide. In the 3 vs. 3, players were distributed in teams of three players plus a goalkeeper (GK+3 vs. 3+GK), while in the 6 vs. 6 the distribution consisted of six players for each team plus a goalkeeper (GK+6 vs. 6+GK). Actions performed by goalkeepers were not assessed or considered for analysis. Prior to the start of each test session, players were informed about the objectives of such tests and were given 30 seconds in order to familiarize with test procedures. All players wore numbered vests in order to be easily identified during video analysis.

### Instrument

We used the System of Tactical Assessment in Soccer (FUT-SAT) (Teoldo et al., 2011a; Teoldo et al., 2010b), which enables the assessment of tactical actions performed by players with and without ball possession. Such assessment is based on ten core tactical principles of soccer with five offensive principles - (i) Penetration; (ii) Offensive Coverage; (iii) Width and Length; (iv) Depth Mobility; (v) Offensive Unity - and five defensive principles - (vi) Delay; (vii) Defensive Coverage; (viii) Balance; (ix) Concentration; (x) Defensive Unity (Teoldo et al., 2009; Worthington, 1974). FUT-SAT comprises two Macro-Categories, seven categories and 76 variables that are organized according to the type of information dealt with by the system (Chart 1). The Macro-Category “Observation” involves three categories and 24 variables: the category “Tactical Principles” includes ten variables; the category “Place of Action on the Playing Field” encompasses four variables; and the category “Action Outcomes” contains ten variables.

### Material

**Figure 1:**
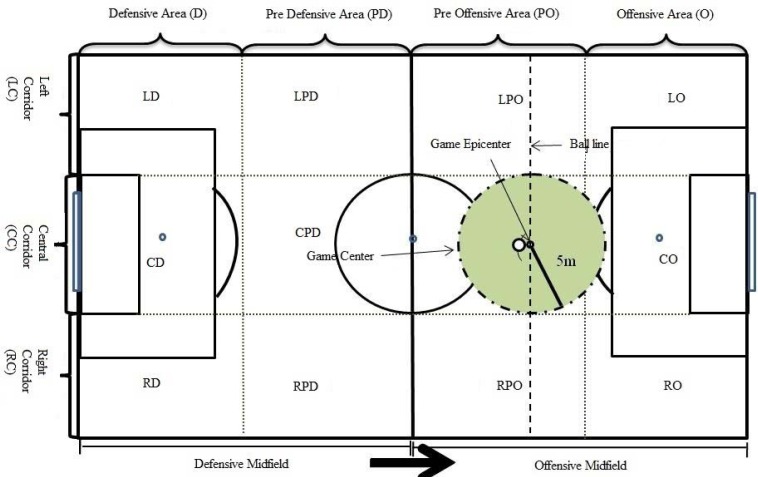
Spatial references used in FUT-SAT’s field test (Teoldo et al., 2011a)

To record field tests, a digital video camera was used (Panasonic^®^ NV-DS35EG). Video footage was introduced in digital format on a laptop (Positivo^®^ Mobile Z65, Intel^®^ Celeron^®^ 540 Processor) via a USB cable and converted to .avi video format. Video processing and analysis were performed through Soccer Analyser^®^ (Picture 1) software. This software was developed for use with FUT-SAT and enables the insertion of spatial references (Figure 1) and also the accurate verification of position and movement of the players, as well as the analysis and categorization of the actions that are to be assessed.

### Statistical Analysis

We performed descriptive analysis (frequency and percentage) for the variables within the categories “Tactical Principles”, “Place of Action on the Playing Field”, and “Action Outcome”. Pearson chi-squared test (χ^2^) was conducted to compare the frequency of tactical actions performed by players in both 3 vs. 3 and 6 vs. 6 situations and significance level was set at *p*<0.05 (O’Donoghue, 2012). For a standardized measure of the extent of the observed effect (Field, 2013), effect sizes (ω) exerted by the variables over the model were obtained through the utilization of the following equation (Cohen, 1992):

$$\omega =\sqrt{\frac{\chi^{2}}{n}}$$

For analysis of effect sizes, we considered the classification proposed by Cohen (1992), who defines their values as small (ω=0.1 or 1% of total variance), medium (ω=0.3 or 9% of total variance) and large (ω=0.5 or 25% of total variance).

Standardized residuals (e) were used to determine which variable(s) in each category contributed most to the value of χ^2^, within both situations (Agresti and Finlay, 2008; Teoldo et al., 2012). Values of standardized residuals were calculated through the utilization of the following equation (Haberman, 1973):

$$e=\frac{O-E}{\sqrt{E}}$$

Cells which contained values of standardized residual that were higher than 2 (e>2), were considered influent for the model (Field, 2013).

For statistical procedures we utilized the software SPSS (Statistical Package for Social Sciences) for Windows^®^ version 18.0.

#### Reliability Analysis

We performed test-retest reliability for the observations, respecting a 20-day interval for reanalysis, thus avoiding task familiarity issues (Robinson and O’Donoghue, 2007). For calculation of reliability, the Cohen’s Kappa test was used. Analyses were verified through the reassessment of 417 tactical actions, or 12% of the overall sample, a value which is greater than the percentage (10%) suggested by literature (Tabachnick and Fidell, 2012). Intra- and inter-observer reliabilities displayed Kappa values of 0.86 (SE=0.032) and 0.84 (SE=0.007), respectively. These values are classified as “Almost Perfect” (0.81 – 1.00) by literature (Landis and Koch, 1977).

## Results

### Tactical Principles

In this category, within the offensive phase, it is possible to observe (Table 1) that the principles of “Penetration” (χ^2^=5.48; *p*=0.19) and “Depth Mobility” (χ^2^=20.18; *p*<0.001) were significantly more frequent in the 3 vs. 3 compared to the 6 vs. 6, while “Offensive Unity” (χ^2^=11.32; *p*=0.001) occurred significantly more times in the 6 vs. 6 than in the 3 vs. 3. During the defensive phase, the principles of “Delay” (χ^2^=10.62; *p*=0.001) and “Defensive Unity” (χ^2^=24.12; *p*<0.001) occurred more times in the 3 vs. 3 situation than in the 6 vs. 6. Conversely, “Defensive Coverage” (χ^2^=19.21; *p*<0.001) and “Balance” (χ^2^=12.21; *p*<0.001) were more frequently performed in the 6 vs. 6 in comparison with 3 vs. 3.

**Figure 3:**
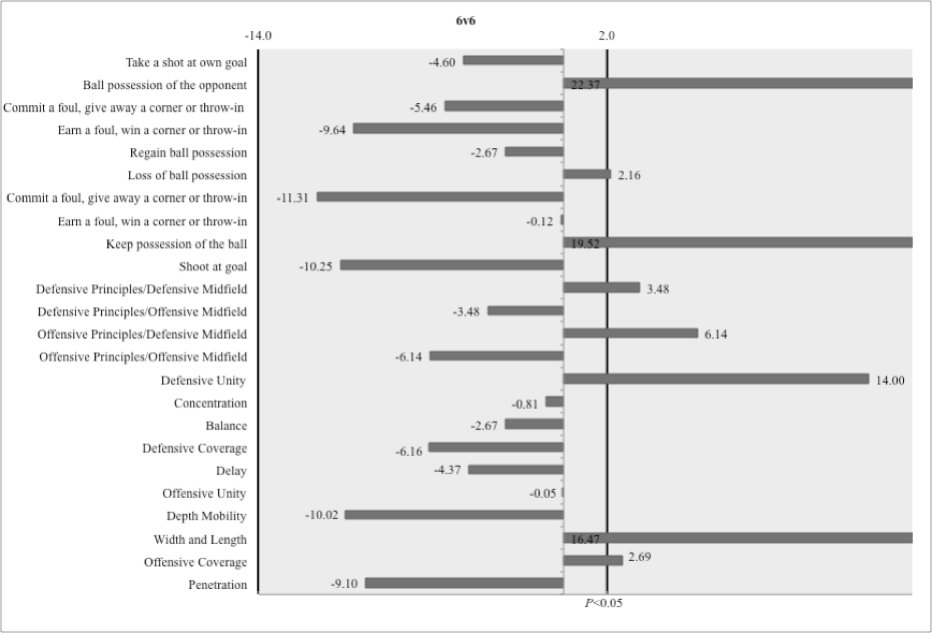
Graphical representation of standardized residuals of variables of 6 vs. 6 SSGs

According to Table 2, values of standardized residuals demonstrated that in the 3 vs. 3 situation during the offensive phase, the actions regarding the tactical principle of “Width and Length” (e=15.33) were significantly more frequent than actions related to other tactical principles, while in the 6 vs. 6, “Offensive Coverage” (e=2.69) and “Width and Length” (e=16.47) presented significantly higher values of standardized residuals than other offensive principles (Figures 3 and 4).

In the defensive phase, “Defensive Unity” displayed higher values of standardized residuals among all tactical principles, in the 3 vs. 3 (e=21.95) and 6 vs. 6 (e=14.00).

### Place of Action on the Playing Field

According to Table 1, defensive actions performed in Offensive Midfield (χ^2^=18.05; *p*<0.001) were significantly more frequent in the 3 vs. 3 than in the 6 vs. 6.

Also, offensive actions performed in “Defensive Midfield” presented significantly higher frequency in the 3 vs. 3 (e=4.39) and 6 vs. 6 (e=6.13), in comparison with those performed in “Offensive Midfield” (Table 2). On the other hand, defensive actions performed in “Defensive Midfield” presented a significant standardized residual only in the 6 vs. 6 (e=3.48), while in the 3 vs. 3 residuals were not statistically significant (e=±0.18).

### Action Outcome

According to Table 1, in the offensive phase, the variables “Shoot at goal” (χ^2^=32.11; *p*<0.001), “Earn a foul, win a corner or throw-in” (χ^2^=5.45; *p*=0.020) and “Commit a foul, give away a corner or throw-in” (χ^2^=27.04; *p*<0.001) presented significantly higher occurrence in the 3 vs. 3 in comparison with the 6 vs. 6. On the other hand, the 6 vs. 6 situation presented higher frequency values for the variables “Keep possession of the ball” (χ^2^=10.30; *p*=0.001) and “Loss of ball possession” (χ^2^=27.99; *p*<0.001). During the defensive phase, “Earn a foul, win a corner or throw-in” (χ^2^=14.00; *p*<0.001) and “Commit a foul, give away a corner or throw-in” (χ^2^=67.85; *p*<0.001) proved to occur more frequently in the 3 vs. 3, while frequency values for “Ball possession of the opponent” (χ^2^=7.12; *p*=0.008) were significantly higher in the 6 vs. 6 situation. In particular, the outcome “Commit a foul, give away a corner or throw-in” within the offensive phase, was the only variable among all, including those from other categories, that displayed effect size value that can be classified as “large” (ω=0.520).

In the 3 vs. 3 situation, “Keep possession of the ball” (e=13.10) and “Earn a foul, win a corner or throw-in” (e=3.65) were significantly more frequent than other variables within the offensive phase (Table 2). In the 6 vs. 6, “Keep possession of the ball” (e=19.51) and “Loss of ball possession” (e=2.16) displayed significantly higher values of standardized residuals than the remainder of the outcomes during the offensive phase. In the defensive phase, “Commit a foul, give away a corner or throw-in” (e=4.44) and “Ball possession of the opponent” (e=13.85) were the most frequent outcomes in 3 vs. 3 SSGs. In the 6 vs. 6, only “Ball possession of the opponent” (e=22.37) presented a significant value of standardized residual among all variables in this phase of the game.

## Discussion

This study aimed to compare players’ tactical behaviour in 3 vs. 3 and 6 vs. 6 soccer small-sided games (SSGs).

Findings imply that, concerning the performance of offensive tactical principles, actions of Penetration and Depth Mobility were significantly more frequent in the 3 vs. 3 than in the 6 vs. 6 SSGs. These results allow us to assume that when fewer players are engaged in the game, more actions involving the rupture of defensive lines and 1 vs. 1 duels are expected. This inference is in accordance with current research that revealed a significantly higher distance covered in sprinting and also a higher number of duels in 4 vs. 4 SSGs in comparison with 11-a-side match play (Dellal et al., 2012). Conversely, 6 vs. 6 SSGs displayed a significantly higher frequency of actions of Offensive Unity than the 3 vs. 3. Such finding suggests that in the 6 vs. 6 SSGs during the offensive phase, players show a higher tendency to position themselves far from the centre of play compared to the 3 vs. 3, probably due to the increase of field size and of the number of players available to receive the ball closer to the centre of play.

In the defensive phase, actions of Delay and Defensive Unity were significantly more frequent in the 3 vs. 3 than in 6 vs. 6 SSGs, while the 6 vs. 6 displayed a higher occurrence of Defensive Coverage and Balance in comparison with the 3 vs. 3. These data might suggest that in the 3 vs. 3, players opted for either a direct duel with the opponent in possession, trying a faster recovery of the ball, or to maintain a safer defensive approach by positioning themselves far away from the centre of play, thus reducing game pace and providing the team with more time to regroup. Inversely, in the 6 vs. 6 players chose a safer behaviour in defense, especially considering the actions of Balance, that indicate that 6 vs. 6 SSGs enable players to stay closer to the centre of play and at the same time to safeguard their own defensive midfield by having more time for decision-making, due to increased field dimensions, that probably forced opponents to cover greater distances in attack (Little and Williams, 2007).

Considering the location on the field where tactical actions took place, results demonstrated that defensive actions in the offensive midfield occurred significantly more often in the 3 vs. 3 than in 6 vs. 6 SSGs, implying that in 3 vs. 3 players appear to have a more aggressive approach when not in possession, by performing actions that aim at the recovery of the ball in the opponent’s half (Silva et al., 2011). This behaviour can be possibly related to the reduced field size, which might have helped the defensive team in limiting the space and time available for the opponent to progress with the ball, thus promoting more changes of ball possession among opposite players in their own defensive midfield (Teoldo et al., 2011b).

Taking into account the outcomes of the tactical actions assessed, the most significant results in the offensive phase concern the variables “Shoot at goal” (ω=0.472) and “Commit a foul, give away a corner or throw-in” (ω=0.520), that displayed significantly higher frequency values in the 3 vs. 3 compared to 6 vs. 6 SSGs. This indicates that when players are in possession, actions are more likely to end in a goal attempt or in loss of ball possession through a foul, corner-kick or throw-in, thus providing the opponent with the opportunity to restart the game through a set play. Equally, in defensive phase, 3 vs. 3 SSGs displayed a higher frequency of the variable “Commit a foul, give away a corner or throw-in” (ω=0.443) and “Earn a foul, win a corner or throw-in” (ω=0.333), what implies that defensive sequences also tend to end up in fouls, corners or throw-ins. Such results allow us to infer that when space is limited (as in 3 vs. 3 SSGs), the game requires players to act more quickly, due to the constraints related to the limited playing area. Therefore, in the offensive phase, whenever actions did not end in a goal attempt, teams which performed such actions and lost possession subsequently, possibly played more assertive defensive styles, that involve trying to recover the ball as quickly as possible, what might have caused more fouls, due to this more aggressive approach (Dellal et al., 2012).

Regarding players’ behaviours within each of the situations (3 vs. 3 and 6 vs. 6 SSGs), the main differences between them are related to the categories “Place of Action on the Playing Field” and “Action Outcome”. Within the category “Place of Action on the Playing Field”, defensive tactical actions performed in the defensive and in the offensive midfield did not reveal any significant difference in the 3 vs. 3 situation. Conversely, defensive actions performed in the defensive midfield were significantly more frequent (e=3.48) than defensive actions performed in the offensive midfield (e=−3.48), what suggests that when more players are involved and field size is larger (as in 6 vs. 6 SSGs), players tend to opt for defensive behaviours that involve marking the opposing team in their own defensive half, thus revealing certain insecurity in marking higher on the field and providing the opposition with space behind the defensive line. The category “Action Outcome” also exhibited substantial results, as the variable “Commit a foul, give away a corner or throw-in” proved to be significantly more frequent than the other variables within the same category only in the 3 vs. 3 SSGs (e=4.44). This finding suggests that challenges for the ball are probably more common in the 3 vs. 3 than in 6 vs. 6 SSGs and corroborates other results within this study that indicate that players often chose a more aggressive approach in the 3 vs. 3 and a safer one in the 6 vs. 6 SSGs (Silva et al., 2011). Therefore, with respect to this inference, it is reasonable to assume that in the processes of teaching, learning and training, coaches should consider increasing the number of players of SSGs gradually, respecting the time players seem to take to adapt to situations involving a larger playing area and more participants than they are familiarized with (Jones and Drust, 2007).

Findings within this study can aid coaches and/or teachers in the sense that the utilization of either SSGs (3 vs. 3 and 6 vs. 6) depends on the purpose of each training session (Köklü, 2012). From the physical/physiological perspective, SSGs with fewer players (as 3 vs. 3) and smaller area size would indicate a predominance of anaerobic metabolism during training sessions, and enable the coverage of longer distances while sprinting and high intensity running compared to small-sided drills that include a higher number of players and larger area size, as 6 vs. 6 games. In contrast, SSGs with a higher number of players provide a more aerobic-related activity (less intense), promoting faster utilization of lactate (Dellal et al., 2012). In technical/tactical terms, the 3 vs. 3 SSGs would enable more 1 vs. 1 challenges and, due to a limited playing area, less time for decision-making, being also more demanding with respect to cognitive skills (Dellal et al., 2011b).

## Conclusions and practical implications

Previous research regarding soccer small-sided games has not compared tactical behaviour in different arrangements. Overall, considering the results and the interpretations within this study, it was possible to verify that players behaved more aggressively in the 3 vs. 3 and more safely in the 6 vs. 6 SSGs, possibly due to the limitations in the available space proper of the 3 vs. 3 configuration. Thus, space management was apparently better in 3 vs. 3 SSGs, probably due to the lower complexity of this arrangement, which involves fewer interactions between the player, his teammates and opponents. These findings can help coaches in better selecting the type of SSG drills according to the purpose of the training session, with respect to players’ tactical development. Future research should consider different youth levels, in order to ascertain whether such behaviours are similar in players of different ages and sports level.

## Figures and Tables

**Figure 2:**
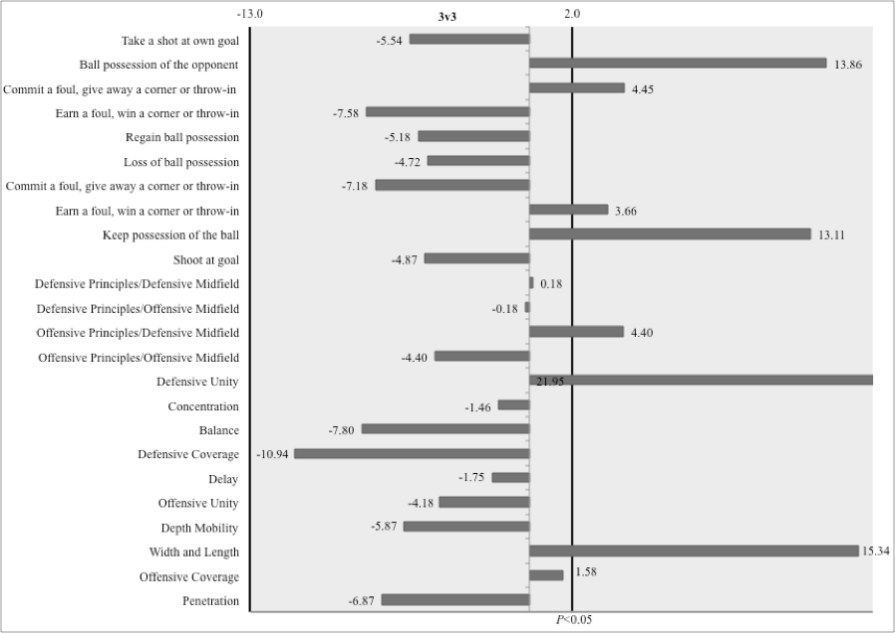
Graphical representation of standardized residuals of variables of 3 vs. 3 SSGs

**Picture 1:**
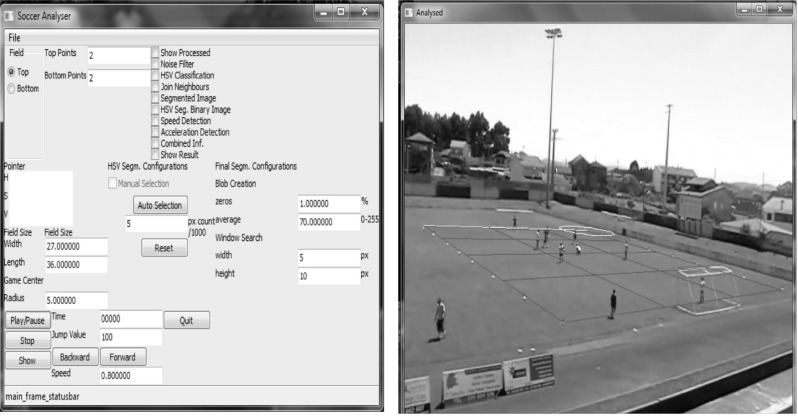
Soccer Analyser^®^ software and spatial references incorporated to test video

**Table 1:** Frequencies, chi-squared (χ^2^) and effect size (ω) values of tactical actions in 3 vs. 3 and 6 vs. 6 SSGs.

**Categories and variables**	**3 vs. 3**	**6 vs. 6**			
	N	N	χ^2^	*p*	*ω*
TACTICAL PRINCIPLES					
*Offensive*					
Penetration	80 (4.48%)	53 (3.13%)	5.48	0.019^*^	0.203
Offensive Coverage	190 (10.63%)	208 (12.27%)	0.81	0.367	0.045
Width and Length	369 (20.65%)	389 (22.95%)	0.52	0.468	0.026
Depth Mobility	93 (5.20%)	41 (2.42%)	20.18	<0.001^*^	0.388
Offensive Unity	115 (6.44%)	172 (10.15%)	11.32	0.001^*^	0.199
*Defensive*					
Delay	164 (9.18%)	110 (6.49%)	10.62	0.001^*^	0.197
Defensive Coverage	38 (2.13%)	87 (5.13%)	19.21	<0.001^*^	0.392
Balance	81 (4.53%)	132 (7.79%)	12.21	<0.001^*^	0.239
Concentration	168 (9.40%)	156 (9.20%)	0.44	0.505	0.036
Defensive Unity	489 (27.36%)	347 (20.47%)	24.12	<0.001^*^	0.170
PLACE OF ACTION ON THE PLAYING FIELD					
*Offensive*					
Offensive Midfield	333 (18.63%)	304 (17.94%)	1.32	0.251	0.045
Defensive Midfield	514 (28.76%)	559 (32.98%)	1.87	0.170	0.041
*Defensive*					
Offensive Midfield	466 (26.08%)	345 (20.35%)	18.05	<0.001^*^	0.149
Defensive Midfield	474 (26.53%)	487 (28.73%)	0.17	0.675	0.013
ACTION OUTCOME					
*Offensive*					
Shoot at goal	106 (5.93%)	38 (2.24%)	32.11	<0.001^*^	0.472
Keep possession of the ball	340 (19.03%)	429 (25.31%)	10.30	0.001^*^	0.116
Earn a foul, win a corner or throw-in	217 (12.14%)	171 (10.09%)	5.45	0.020^*^	0.119
Commit a foul, give away a corner or throw-in	76 (4.25%)	24 (1.42%)	27.04	<0.001^*^	0.520
Loss of ball possession	108 (6.04%)	201 (11.86%)	27.99	<0.001^*^	0.301
*Defensive*					
Regain ball possession	117 (6.55%)	132 (7.79%)	0.90	0.342	0.060
Earn a foul, win a corner or throw-in	84 (4.70%)	42 (2.48%)	14.00	<0.001^*^	0.333
Commit a foul, give away a corner or throw-in	249 (13.94%)	96 (5.66%)	67.85	<0.001^*^	0.443
Ball possession of the opponent	378 (21.15%)	455 (26.84%)	7.12	0.008^*^	0.092
Take a shot at own goal	112 (6.27%)	107 (6.31%)	0.11	0.735	0.022
**Total**	1,787	1,695			

* p<0.05 (Significant difference between 3 vs. 3 and 6 vs. 6 SSGs)

**Table 2:** Standardized residuals (e) of tactical actions in 3 vs. 3 and 6 vs. 6 small-sided games (SSGs).

**Categories and variables**	**Standardized Residuals (e)**	
	**3 vs. 3**	**6 vs. 6**
TACTICAL PRINCIPLES		
*Offensive*		
Penetration	−6.86	−9.10
Offensive Coverage	1.58	2.69^*^
Width and Length	15.33^*^	16.47^*^
Depth Mobility	−5.87	−10.01
Offensive Unity	−4.18	−0.04
*Defensive*		
Delay	−1.75	−4.37
Defensive Coverage	−10.94	−6.15
Balance	−7.80	−2.66
Concentration	−1.45	−0.80
Defensive Unity	21.95^*^	14.00^*^
PLACE OF ACTION ON THE PLAYING FIELD		
*Offensive*		
Offensive Midfield	−4.39	−6.13
Defensive Midfield	4.39^*^	6.13^*^
*Defensive*		
Offensive Midfield	−0.18	−3.48
Defensive Midfield	0.18	3.48^*^
ACTION OUTCOME		
*Offensive*		
Shoot at goal	−4.87	−10.24
Keep possession of the ball	13.10^*^	19.51^*^
Earn a foul, win a corner or throw-in	3.65^*^	−0.12
Commit a foul, give away a corner or throw-in	−7.17	−11.31
Loss of ball possession	−4.71	2.16^*^
*Defensive*		
Regain ball possession	−5.17	−2.66
Earn a foul, win a corner or throw-in	−7.58	−9.64
Commit a foul, give away a corner or throw-in	4.44^*^	−5.45
Ball possession of the opponent	13.85^*^	22.37^*^
Take a shot at own goal	−5.54	−4.60

* Significant standardized residuals (e>2)

**Chart 1:** Definitions, categories and sub-categories of variables assessed by FUT-SAT

**Categories**	**Sub-Categories**	**Variables**	**Definitions**
Tactical Principles	Offensive	Penetration	Movement of player with the ball towards the goal line.
		Offensive Coverage	Offensive supports to the player with the ball.
		Depth Mobility	Movement of players between the last defender and goal line.
		Width and Length	Movement of players to extend and use the effective play-space.
		Offensive Unity	Movement of the last line of defenders towards the offensive midfield, in order to support offensive actions of the teammates.
	Defensive	Delay	Actions to slow down the opponent’s attempt to move forward with the ball.
		Defensive Coverage	Positioning of off-ball defenders behind the “delay” player, providing defensive support.
		Balance	Positioning of off-ball defenders in reaction to movements of attackers, trying to achieve the numerical stability or superiority in the opposition relationship.
		Concentration	Positioning of off-ball defenders to occupy vital spaces and protect the scoring area.
		Defensive Unity	Positioning of off-ball defenders to reduce the effective play-space of the opponents.
Place of Action	Offensive Midfield	Offensive Actions	Offensive actions performed in the offensive midfield.
		Defensive Actions	Defensive actions performed in the offensive midfield.
	Defensive Midfield	Offensive Actions	Offensive actions performed in the defensive midfield.
		Defensive Actions	Defensive actions performed in the defensive midfield.
Action Outcome	Offensive	Shoot at goal	When a player shoots at goal, and (a) scores a goal, (b) the goalkeeper makes a save, (c) the ball touches one of the goalposts or the crossbar.
		Keep possession of the ball	When team players execute passes to each other and keep up with the ball.
		Earn a foul, win a corner or throw-in	When the match is stopped due to a foul, corner or throw-in; the team that was attacking KEEPS possession of the ball.
		Commit a foul, give away a corner or throw in	When the match is stopped due to a foul, corner or throw-in; the possession of the ball CHANGES to the team that was in defence.
		Loss of ball possession	When the attacking team loses the ball possession.
	Defensive	Regain the ball possession	When the defensive players regain the ball possession.
		Earn a foul, win a corner or throw-in	When the match is stopped due to a foul, corner or throw-in and the possession of the ball CHANGES to the team that was in defence.
		Commit a foul, give away a corner or throw in	When the match is stopped due to a foul, corner or throw-in; the team that was attacking KEEPS possession of the ball.
		Ball possession of the opponent	When the defensive players do not regain the ball possession.
		Take a shot at own goal	When the defensive team takes a shot at their own goal, and (a) takes a goal, (b) the goalkeeper makes a save, (c) the ball touches one of the goalposts or the crossbar.
